# People with dementia and informal caregivers’ perceptions of barriers and facilitators to implementing a behavioral activation intervention: a qualitative study using Normalization Process Theory

**DOI:** 10.1186/s12877-025-06087-1

**Published:** 2025-06-11

**Authors:** Frida Svedin, Oscar Blomberg, Anders Brantnell, Paul Farrand, Louise von Essen, Anna Cristina Åberg, Joanne Woodford

**Affiliations:** 1 Department of Women’s and Children’s Health, CIRCLE – Complex Intervention Research in Health and Care, Uppsala University, Uppsala, 751 85 Sweden; 2 Department of Civil and Industrial Engineering, Industrial Engineering and Management, Uppsala University, Uppsala, 751 21 Sweden; 3 Development and Research (CEDAR); Psychology, Clinical Education, University of Exeter, Perry Road, Devon, EX4 4QG United Kingdom; 4 Medical Sciences, School of Health and Welfare, Dalarna University, Falun, 791 88 Sweden

**Keywords:** Dementia, Mental health, Depression, Implementation potential, Normalization process theory

## Abstract

**Background:**

While people with dementia commonly experience depression, access to psychological treatment is low. A guided low-intensity behavioral activation intervention may represent a solution. Considering implementation barriers and facilitators is important during intervention development to increase the future implementation potential of an intervention. However, involving patients and informal caregivers in identifying implementation barriers and facilitators during intervention development is rarely done. Involving patients and informal caregivers may be particularly important for low-intensity interventions, given the active role they are expected to adopt for successful intervention implementation, e.g., they are seen as active agents as opposed to passive recipients. Study aims were to: (1) develop an understanding of the existing healthcare and community support in the Swedish context for people with dementia and informal caregivers, and (2) identify people with dementia and informal caregivers’ perceived barriers and facilitators to intervention uptake informed by Normalization Process Theory (NPT).

**Methods:**

Following an interview guide informed by NPT, semi-structured interviews were held with people with dementia (*n* = 8) and informal caregivers (*n* = 19). Interview transcripts were analyzed using the NPT coding manual, with an inductive approach adopted for data related to the core theoretical NPT constructs, but not represented within its sub-constructs.

**Results:**

Nine deductive and three inductive categories related to three core NPT constructs (*Coherence, Cognitive Participation, Collective Action*) were identified. Barriers included: (1) extensive intervention material, (2) dementia symptoms, and (3) lacking acceptance of the dementia. Facilitators included: (1) intervention having potential to fill a large psychological treatment gap, (2) understanding and agreeing upon the intervention purpose and potential benefits, (3) intervention guide having professional experience in dementia, and (4) a trusting face-to-face relationship with the intervention guide.

**Conclusions:**

Implementation barriers and facilitators were identified alongside evidence-based implementation strategies to be adopted in the future. Additional barriers and facilitators were identified to those recognized by dementia healthcare and community interest-holders, emphasizing the importance of recognizing the potential diversity of perspectives among different groups within the healthcare triad. By integrating these diverse perspectives early in the intervention development, we aim to develop an intervention optimized for implementation within real-world settings, meeting global health priorities to support people with dementia and their families live well with dementia.

**Trial registration:**

Not applicable.

**Supplementary Information:**

The online version contains supplementary material available at 10.1186/s12877-025-06087-1.

## Background

People with dementia commonly experience depression [1], which is associated with functional decline, poor quality of life, and reduced activities of daily living [2]. Policies to support people with dementia remain living in the community [3], alongside reductions in formal care service provision, are resulting in an increased reliance on informal caregivers [4]. However, informal caregivers themselves may experience depression, anxiety, and stress [5]. Given the person with dementia and informal caregiver can influence each other’s psychological well-being, it is essential both dyad members function well [6].

Supporting people with dementia and their informal caregivers to live well with dementia is a global health priority [7]. Interventions to support people with dementia and depression are particularly important given approximately 39% experience depression [8]. Although evidence-based psychological interventions can be effective for people with dementia and depression [1], access remains limited. Barriers to accessing psychological interventions among people with dementia include stigma towards dementia and mental health in older people, limited resources, and high work pressure on healthcare professionals [9].

One solution to improve access to psychological interventions for people with dementia is via guided low-intensity behavioral activation (BA) [10]. Low-intensity BA is delivered in a self-help format via written workbooks, smartphone applications, or the internet with guidance from trained healthcare professionals. BA is an evidence-based psychological intervention for depression targeting behavioral avoidance, a mechanism identified as leading to depression. Low-intensity BA uses a simple, structured, and graded approach to support people with dementia re-engage with necessary, routine, and pleasurable activities they have stopped doing [11]. Improved functioning in people with dementia is also associated with decreased burden and improved well-being in informal caregivers [12].

Given the promise of guided low-intensity BA, an intervention was developed and tested [13, 14] in the United Kingdom following the Medical Research Council complex interventions framework [15]. A novel aspect of the intervention relates to involving informal caregivers, with guidance provided by a trained healthcare professional, to support the person with dementia to engage with and use the intervention [10]. However, before adopting the intervention in Sweden, adapting it alongside key interest-holders to maximize acceptability [16] while maintaining fidelity to the evidence-based components of BA [17] is required. To maximize acceptability and contextual fit with the Swedish context, initial intervention development and adaptation work have been conducted alongside people with dementia, informal caregivers, healthcare professionals, and community interest-holders [18]. Barriers and facilitators to implementation have been identified by healthcare professionals and community interest-holders (hereafter referred to as ‘professionals’) [19] to increase future implementation potential.

Given that guided low-intensity psychological interventions may be considered a form of self-management, this intervention aligns with global healthcare shifts from hospital-centered to integrated care [20] by supporting people with dementia to play an active role in managing their condition. Involving patient-caregiver dyads during intervention development may increase the implementation potential of self-management interventions [21]. Involvement is especially important for people with dementia who have historically been excluded from qualitative research, with their voices at an increased risk of being suppressed and ignored [22]. Further, in accordance with the Medical Research Council complex interventions framework, involving key interest-holders in intervention development is essential to facilitate our understanding of factors related to future implementation. This includes identifying and addressing implementation barriers [15] and enhancing intervention acceptability and feasibility [23]. Embedding consideration of implementation across all research phases (including the development of complex interventions) can increase the likelihood of implementing effective and accessible mental health interventions into real-world settings [24]. Taking implementation into consideration, e.g., by assessing implementation potential in the early intervention development phase, may not only minimize research waste but potentially accelerate intervention uptake into clinical practice [25].

Normalization Process Theory (NPT) is an implementation theory that can facilitate understanding of implementation barriers and facilitators and increase implementation potential [26]. It focuses on the individual and collective work interest-holders must do to implement complex interventions in routine practice [27] and includes four core theoretical constructs: *Coherence*, *Cognitive Participation*, *Collective Action*, and *Reflexive Monitoring* [26]. NPT has been widely used to explore professionals’ perspectives on implementation, e.g., [28, 29], and some studies have used it to explore people with dementia and informal caregivers’ perspectives on implementation, e.g., [30]. However, to date, no studies have explored the implementation potential of psychological interventions for people with dementia and depression from the perspectives of people with dementia and informal caregivers.

### Aim

This study is part of a wider series of studies [10] with the overall aim of adapting a guided low-intensity BA intervention for people with dementia and depression and their informal caregivers, and enhance future implementation potential for the Swedish context. In this study, we use NPT to develop an understanding of barriers and facilitators to implementing a guided low-intensity BA intervention from the perspectives of people with dementia and informal caregivers.

Specific objectives were to: (1) develop an understanding of the existing healthcare and community support in the Swedish context for people with dementia and informal caregivers, and (2) identify people with dementia and informal caregivers’ perceived barriers and facilitators to intervention uptake informed by NPT.

## Methods

### Qualitative approach and research paradigm

We adopted pragmatism as the qualitative approach and research paradigm.

### Researcher characteristics and reflexivity

FS, a female doctoral candidate (MSc Public Health), conducted the analysis. FS was supervised by ACÅ and has attended postgraduate qualitative methods training. ACÅ is a female professor (Medical Science), focusing on geriatrics and implementation science. She led the development of the Swedish version of the NPT outcome measurement, with extensive qualitative research experience. JW is a female assistant professor (Healthcare Sciences) and the principal investigator. She has extensive experience in conducting qualitative research and led the peer examination discussions. Additional peer examination was provided by AB, a male assistant professor (Industrial Engineering and Management), OB, a male doctoral candidate (MSc Public Health), LvE, a female professor (Healthcare Sciences), and PF, a male professor (Evidence-Based Psychological Practice). All have been part of the research team since study setup.

### Study design

Qualitative study using semi-structured interviews. Reporting follows the Standards for Reporting Qualitative Research [31] (Additional file 1).

### Context

In Sweden, approximately 160,000 people are living with dementia [32]. Responsibility for general healthcare resides with Sweden’s 21 regions, while social and home care resides with Sweden’s 290 municipalities [33]. In 2017, Sweden began to implement “God och nära vård” (Good and Close Care), moving from hospital-centered to integrated care, with the main objective of increasing patients’ involvement in their healthcare [34].

### Study participants

Eligible people with dementia were adults: (1) with a self-reported diagnosis of dementia, (2) living at home, (3) capacity to provide informed consent, and (4) able to speak and understand Swedish. Eligible informal caregivers were adults: (1) who self-identified as an informal caregiver of a person with dementia, and (2) able to speak, understand, and write in Swedish. Exclusion criteria for people with dementia and informal caregivers were: (1) self-reported diagnosis of a severe and enduring mental health difficulty, (2) visual or auditory impairment that would hinder participation in an interview and/or providing intervention feedback, and/or (3) self-reported misuse of alcohol, or prescription or street drugs. Given people with dementia and informal caregivers only reviewed the intervention and its material, rather than receiving the intervention, they did not need to be in dyadic pairs to participate in this study (i.e., one dyad member could participate without the other).

### Recruitment

People with dementia and informal caregivers were recruited across Central Sweden (Uppsala, Stockholm, Västmanland, Södermanland, and Örebro) via memory clinics, health and social care services, social media, and day care centers (only informal caregivers) from September 2021 to January 2022. We asked recruitment site staff to provide brief verbal study information and a study invitation pack to interested people with dementia and informal caregivers. Those who were interested were asked to contact the research team via e-mail or telephone or return a reply slip by post.

### Informed consent and eligibility screening

FS or OB assessed people with dementia for capacity to provide consent following the Swedish Health and Medical Services Act (2017:30). Written informed consent was obtained from all people with dementia and informal caregivers. Thereafter, an eligibility screen was conducted, and eligible participants completed a background questionnaire including sociodemographic characteristics and experience of problems with psychological well-being.

### Reasons for non-participation

People with dementia and informal caregivers declining participation (*n* = 10) were invited to fill out an anonymous reason for non-participation form. The form included a closed multiple-choice question and a free-text response option. Reasons for non-participation are reported elsewhere [18].

### Intervention

**Fig. 1:**
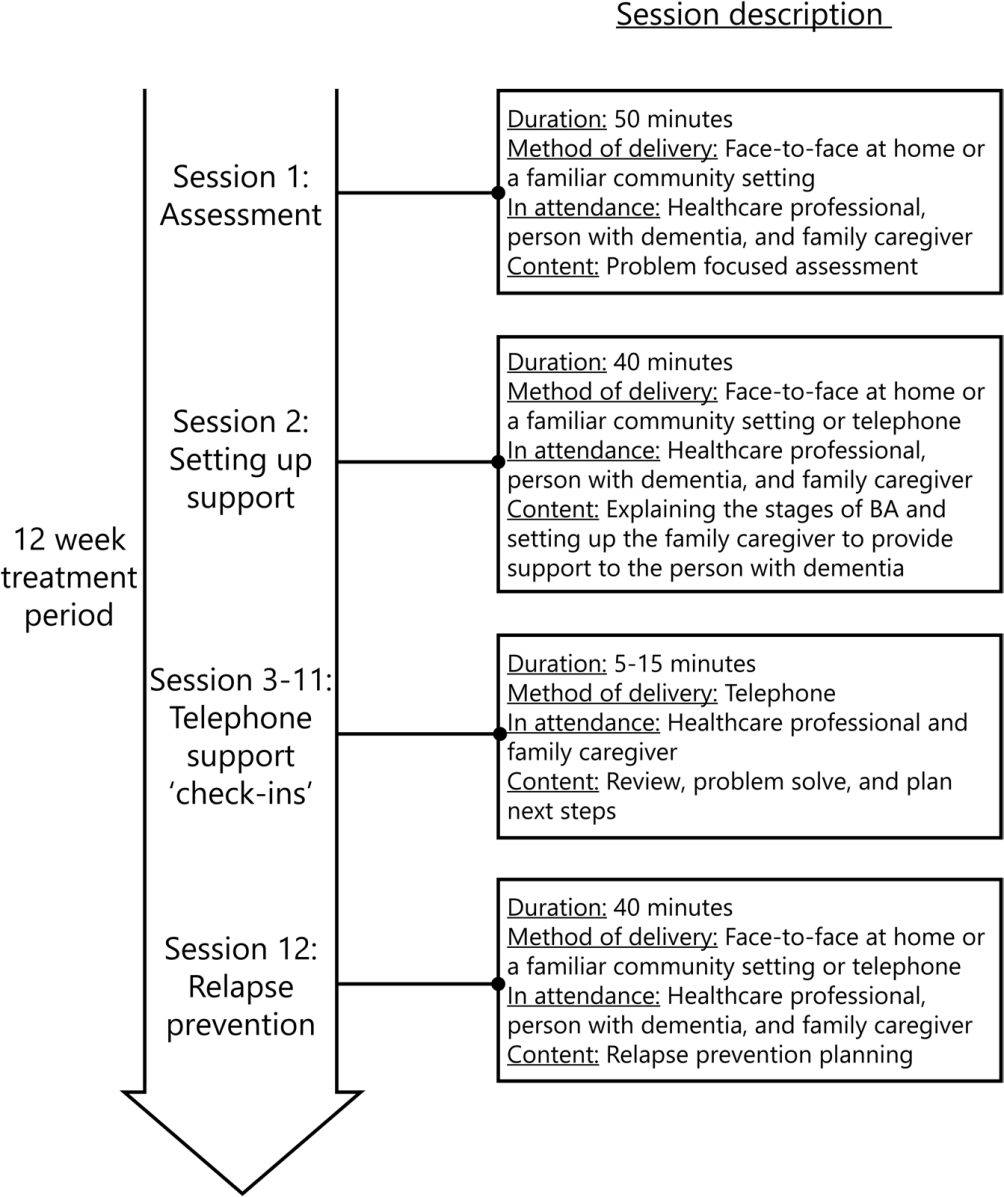
Delivery model of the guided low-intensity behavioral activation intervention developed in the United Kingdom. (Adapted from [19] with permission to use under the terms of the Creative Commons CC BY 4.0 license)

The clinical protocol developed in the United Kingdom is published elsewhere [14]. The intervention is designed for community-dwelling people with mild-to-moderate dementia experiencing depression symptoms and is based on a simple BA approach [11]. The intervention is delivered via two written workbooks: one for people with dementia and one for informal caregivers. The person with dementia is supported by an informal caregiver to gradually re-engage in pleasurable, necessary, and routine activities they have stopped doing and/or identify new activities with similar meaning, importance, or value. A trained healthcare professional (intervention guide) provides guidance to the dyad. Supervisors provide training and supervision to intervention guides. See Fig. 1 for an overview of the intervention delivery model.

### Data collection

People with dementia (*n* = 8) and informal caregivers (*n* = 19) participated in individual semi-structured interviews from October 2021 to March 2022. Depending on preference, interviews were held face-to-face (*n* = 19) or via telephone (*n* = 8). Before interviews, people with dementia and informal caregivers were provided with a written summary of the intervention delivery model and printed workbooks translated into Swedish to their home address via the post. FS or OB conducted semi–structured interviews (20–85 min) following an interview guide informed by three NPT core constructs: (1) *Coherence*, (2) *Cognitive Participation*, and (3) *Collective Action* (Additional file 2). The core construct *Reflexive Monitoring* was not included, given it refers to how individuals and groups appraise how the intervention affects them in practice, and can be considered less relevant during early intervention development [35]. Examples of interview questions and corresponding NPT core construct are provided in Table 1.

**Table 1:** Example of interview questions and corresponding Normalization Process Theory (NPT) core construct

**NPT core construct**	**Interview question **
Coherence	• What impact do you think the intervention can have on people with memory difficulties?
Cognitive Participation	• What type of support do you think people with memory difficulties need to understand what the intervention is and how it should be used?
Collective Action	• There are currently no trained healthcare group who can provide intervention guidance to informal caregivers and people with memory difficulties in Sweden. Who or which group do you think is best at providing this guidance?

### Participant characteristics

The mean age of people with dementia was 73 years, and four (50%) were female, with six retired, one on sick leave, and one working full-time. All had a self-reported Alzheimer’s diagnosis with a mean time of two years since diagnosis. The majority (75%) lived with someone and received support from an informal caregiver (63%). The majority (75%) self-reported problems with their mood or psychological well-being prior to the last 12 months and/or during the last 12 months (Table 2).

**Table 2:** Sample characteristics of people with dementia

Participant	Time since dementia diagnosis (years)	Age (years)	Self-reported problems with mood or psychological well-being	
			** < 12 months**	** > 12 months**
Person with Dementia 1	Missing	80–84	No	No
Person with Dementia 2	Missing	80–84	No	No
Person with Dementia 3	< 1	55–59	Yes	No
Person with Dementia 4	2	70–74	Yes	Yes
Person with Dementia 5	2	80–84	Yes	Yes
Person with Dementia 6	2–3	60–64	No	Yes
Person with Dementia 7	3	60–64	Yes	Yes
Person with Dementia 8	5	75–79	Yes	No

The majority (63%) of informal caregivers were female, had a mean age of 73 years, and had been in a caregiving role for a mean of 4 years. Most (69%) were retired, and the majority (89%) were partners to a person with dementia and lived with them. The majority (74%) self-reported problems with their mood or psychological well-being prior to the last 12 months and/or during the last 12 months (Table 3).

**Table 3:** Sample characteristics of informal caregivers

Participant	Time caregiving for person with dementia (years)	Age (years)	Self-reported problems with mood or psychological well-being	
			** < 12 months**	** > 12 months**
Informal caregiver 1	Missing	70–74	Missing	Missing
Informal caregiver 2	Missing	80–84	No	Yes
Informal caregiver 3	2	50–54	No	Yes
Informal caregiver 4	2	60–64	No	No
Informal caregiver 5	2	75–79	Yes	No
Informal caregiver 6	2	90–94	Yes	No
Informal caregiver 7	3	60–64	No	No
Informal caregiver 8	3	65–69	Yes	No
Informal caregiver 9	3	65–69	Yes	No
Informal caregiver 10	3	70–74	Yes	Yes
Informal caregiver 11	3	80–84	Yes	No
Informal caregiver 12	4	70–74	Yes	Yes
Informal caregiver 13	4	75–79	Yes	No
Informal caregiver 14	4	80–84	No	No
Informal caregiver 15	5	70–74	No	No
Informal caregiver 16	6	75–79	No	Yes
Informal caregiver 17	7	55–59	Yes	No
Informal caregiver 18	7	75–79	Yes	Yes
Informal caregiver 19	9	70–74	Yes	Yes

### Data processing

An external professional transcriber transcribed interview recordings verbatim. Transcripts were then uploaded into NVivo 14 to organize the data and facilitate the coding process.

### Data analysis

Adopting a deductive coding approach, FS coded data relevant to core NPT constructs to NPT sub-constructs following the NPT coding manual [36]. Codes pertinent to study objectives and relating to core NPT constructs, but unrelated to their sub-constructs, were organized into inductive sub-categories. Within *Coherence*, two sub-constructs (*Communal Specification* and *Individual Specification*) were combined into one (*Specification*) due to challenges in differentiating data referring to communal versus individual specification. Categorization workshops were held with FS and ACÅ (*n* = 2), and FS, ACÅ and JW (*n* = 3). Categories and category descriptions were provided in English to ACÅ and JW for peer examination. Supporting quotations were selected and translated by a native Swedish speaker (FS) and reviewed by a native English speaker (JW).

### Trustworthiness

Trustworthiness was established using peer examination of supervising researchers (ACÅ and JW) and from the wider team (AB, LvE, OB, PF), and record keeping [37]. Disconfirming cases and divergent discourses were discussed with supervising researchers.

### Public contribution in research

A Public Advisory Group of informal caregivers of people with dementia (*n* = 4) worked alongside the research team to: (1) make sense of and interpret findings for the wider project, and (2) co-design intervention workbooks. Public contributors were wives and daughters of people with dementia, aged between 44–71 years, and had 5–9 years’ experience of caring for someone with dementia. The impact of public contribution activities will be reported elsewhere.

## Results

Findings are organized under three core NPT constructs with applicable NPT sub-constructs (deductive sub-categories) and inductive sub-categories. To inform future implementation processes, identified barriers and facilitators, alongside their respective sub-constructs and core NPT constructs, are presented in Tables 4, 5 and 6. Barriers and facilitators perceived by people with dementia and informal caregivers are not compared and contrasted. However, we report disconfirming cases and divergent opinions to enhance understanding and strengthen credibility. Supportive quotations are provided alongside the text.

**Table 4:** Facilitators and barriers mapped to the sub-constructs of the Normalization Process Theory (NPT) core construct *Coherence*

**NPT** **Sub-construct and definition**	**Facilitator**	**Barrier**
*Differentiation* Ability to distinguish the intervention from current practice	• Perceiving the intervention as different from current practice	• Differing provision of support to people with dementia geographically
*Specification* Agreement concerning the intervention purpose	• Agreeing on and understanding the intervention purpose	
*Internalization* Understanding the value, benefits, and importance of the intervention	• Considering the intervention as a structured, motivating, and easy to use tool • Facilitating within-dyad communication • Facilitating more structured support from informal caregivers • Increasing well-being • Maintaining activities of daily living • Motivating the person with dementia • Reducing caregiver burden • Strengthening the informal caregiver role	• Difficulties finding a good balance when engaging with the intervention • Increasing within-dyad conflicts • Increasing stress among people with dementia

**Table 5:** Facilitators and barriers mapped to the sub-constructs of the Normalization Process Theory (NPT) core construct *Cognitive Participation*

NPT Sub-construct and definition	Facilitator	Barrier
*Enrolment* Organizing in order to contribute to the intervention work and to sustain intervention engagement	• Explaining potential positive impacts • Providing a step-by-step and thorough intervention introduction face-to-face • Providing guidance from the intervention guide	• Having reluctancy to change • Including too extensive intervention material • Lacking motivation for taking part in the intervention (people with dementia)
*Legitimation* Agreement that the intervention is the right thing to do and that they can make a valid contribution to it	• Agreeing the intervention is relevant • Being interested in using the intervention • Recognizing the role of both dyad members in the intervention	• Experiencing severe dementia • Lacking interest in intervention participation
*Relational Interaction*^a^ Establishing and maintaining care relationships required for commitment and sustained intervention use	• Building a relationship between intervention guide and dyad • Having motivating and inspiring intervention guides • Trusting the intervention guide	
*Definition and Evaluation of the Situation*^a^ Understanding, defining, and evaluating the dyad’s situation and intervention need	• Accepting the dementia diagnosis • Recognizing a need for support	• Feeling pressured to use the intervention • Unwillingness to use the intervention • Failing to recognize a need for support • Lacking an acceptance of the dementia diagnosis • Lacking incentives due to progressive nature of dementia (informal caregivers)

^a^Inductive category

**Table 6:** Facilitators and barriers mapped to the sub-constructs of the Normalization Process Theory (NPT) core construct *Collective Action*

**NPT** **Sub-construct and definition**	**Facilitator**	**Barrier**
*Interactional Workability* Working with each other, and the intervention, to seek to operationalize it in routine practice	• Facilitating better structure and routines in daily life	• Being overly ambitious working with the intervention
*Relational Integration* Building accountability and maintaining confidence in each other in continued intervention use	• Having confidence in dementia/specialist nurses possessing competence and knowledge to guide the intervention	• Lacking trust in assistant nurses, general practitioners, and volunteers
*Skill-set Workability* Allocating work around a set of practices to operationalize the intervention in routine practice	• Having intervention guides with lived experience of informal caregiving • Having intervention guides with a healthcare background, and knowledge and experience in dementia • Providing education for informal caregivers • Providing training for intervention guides	
*Contextual Integration* Allocating resources, protocols, policies, and procedures to operatize the intervention in routine practice		• Lacking competent staff • Lacking funding • Lacking time (informal caregivers)
*Prerequisites for Intervention Interaction*^a^ Prerequisites for the dyad to manage and undertake the intervention in their everyday life	• Being motivated and disciplined to continue intervention engagement (people with dementia) • Having good within-dyad communication	• Dementia symptoms • Fluctuating symptoms of dementia on a day-to-day basis

^a^Inductive category

### Coherence

Data supporting NPT sub-constructs *Differentiation*, *Specification*, and *Internalization* were identified (Table 4).

#### Differentiation

People with dementia and informal caregivers differentiated the intervention from current practice, perceiving there to be a lack of psychological support for people with dementia in Sweden:

“*I don’t get any support, apart from my family, which I shouldn't belittle in any way because they do a huge job, but I don’t get anything else”* (Person with dementia 3)

Informal caregivers also expressed large unmet support needs, requesting more support instead of being ‘left alone’ after diagnosis. People with dementia and informal caregivers raised that while some support services were available, e.g., caregiver support services, the availability of support services varied geographically. Primary healthcare centers were mentioned as potential sources of support, but the variability in dementia knowledge of staff was emphasized as a barrier:

*“General practitioners get very little education in this*. *In my experience, when you go to a primary healthcare center, they barely have more knowledge than me”* (Informal caregiver 1)

#### Specification

People with dementia and informal caregivers agreed that the overall purpose of the intervention is to support and give structure to the dyad in their everyday life and improve well-being. The intervention was perceived as supporting independence and home-living, and helping people with dementia maintain their ability to perform activities of daily living and increase activity levels:

*“To increase well-being and have a dignified life. To be as active as possible for as long as possible, and to have as much independence and participation as possible”* (Informal caregiver 17)

One person with dementia reflected that the purpose of the intervention was twofold: to support their own well-being and to contribute to helping others in a similar situation:

*“Well, the purpose is… there are really two aspects. The most important one, selfishly speaking, is if I can get some support myself to have a better life and somehow keep functioning. But then also, maybe in some way, to pass something on to others. I mean, I’m not alone in this misery… That it can be developed and turned into something more”* (Person with dementia 7)

#### Internalization

The intervention was perceived as having the potential to impact on people with dementia and informal caregivers positively:

*“When I got this in my hands, I got really happy, a support program… I’ve missed that. You meet people in the same situation and you get* [support] *through the dementia association, but this is something completely different”* (Person with dementia 7)

Perceived positive impacts included motivating the person with dementia, reducing caregiver burden, strengthening caregiver role, structuring caregiver support, and *“better communication”* (Person with dementia 6) within the dyad. One informal caregiver argued that:

*“If the person with dementia experiences increased well-being and feels inspired, it will also benefit the informal caregiver”* (Informal caregiver 4)

The intervention was described as a practical and easy to use tool for informal caregivers that could increase understanding of the person with dementia and:

*“Give the informal caregiver better possibilities for supporting the person with dementia”* (Informal caregiver 10)

Potential negative impacts were also raised, e.g., increasing stress for people with dementia, causing disagreements and within-dyad conflicts, and increasing caregiver burden. Intervention completion was perceived as dependent on balancing caregiving demands and engaging the person with dementia. Some voiced concerns that ‘failing’ or being ‘forced’ to use the intervention may negatively impact engagement.

### Cognitive participation

Data supporting NPT sub-constructs *Enrolment* and *Legitimation* were identified, with no data addressing *Initiation* or *Activation*. Analysis also resulted in two inductive sub-categories: *Relational Interaction,* and *Definition and Evaluation of the Situation* (Table 5).

#### Enrolment

A need to prepare the dyad to adopt and contribute to the intervention by providing step-by-step introduction face-to-face was expressed:

*“Little by little. It’s sheet upon sheet of information, and it might just start to spin and become too much, so rather one piece at a time”* (Person with dementia 3)

A need for the intervention guide to clearly explain the intervention and potential benefits was also voiced:

“*The intervention guide needs to introduce the whole thing, of course. I think you need that, otherwise you would not really understand the point of doing this”* (Informal caregiver 16)

Providing more support during initial intervention steps was considered a facilitator to intervention engagement. Conversely, lack of motivation, reluctance to change, and extensive intervention material were considered barriers.

#### Legitimation

Generally, informal caregivers could see their role in supporting the person with dementia in using the intervention. However, their own physical health and lack of time were perceived as barriers. People with dementia and informal caregivers considered the intervention relevant and that they could make a valid contribution. However, one person with dementia could not see their role in or need for the intervention:

*“I don’t really understand why I would need it*… *I wouldn’t write it down even if I had this in front of me… I don’t have any* [cognitive] *difficulties yet”* (Person with dementia 2)

Interest in using the intervention was raised as an important facilitator for intervention success. Informal caregivers perceived the intervention to fit people with mild-to-moderate dementia as opposed to those with more severe dementia:

*“If he hadn’t been so far into his dementia, this intervention would have been perfect”* (Informal caregiver 14)

#### Relational interaction

Establishing and maintaining a trusting relationship between the dyad and the intervention guide was perceived as important for intervention engagement and continued maintenance. Face-to-face meetings were raised as highly important, especially during the initial stages of working with the intervention:

*“When you start the intervention, I’d like to meet the person*, *so we get contact and feel if it works well with the personal chemistry and everything like that. I don’t think it’s the same talking on the phone”* (Informal caregiver 18)

The importance of meeting face-to-face was also agreed on by people with dementia, expressing that:

*“I think face-to-face meetings. Especially when it comes to this illness… You can sort of see whether I understand or not… I’m old-fashioned that way, I think personal meetings are important”* (Person with dementia 7)

People with dementia and informal caregivers described the intervention guide as essential for engagement, particularly for informal caregivers:

*“The intervention guide is very important as support and vent for the informal caregiver. That’s the relationship you have to build on, and that’s what’s good about this; that you get a supportive contact and can talk to someone once a week, who can also structure the support for the loved one”* (Informal caregiver 15)

#### Definition and evaluation of the situation

Recognition and acceptance of dementia were perceived as prerequisites for intervention use and maintenance:

*“The first thing is for them to accept that they have dementia, that is number one. And if they don’t do that, it will be difficult”* (Informal caregiver 1)

Lack of interest, *“feeling pressured”* (Informal caregiver 5) in engaging with the intervention, or *“unwillingness”* (Person with dementia 5) were also perceived as barriers:

*“If the person* [with dementia] *doesn’t want to* [engage with the intervention],* then it’s difficult”* (Informal caregiver 9)

Lack of incentives among informal caregivers due to the progressive nature of the dementia was also raised as a potential barrier for intervention success:

*“I know he* [person with dementia] *won’t get any better, so it feels pointless in that way”* (Informal caregiver 14)

### Collective action

Data supporting the NPT sub-constructs *Interactional Workability*, *Relational Integration*, *Skill-Set Workability*, and *Contextual Integration* were identified. Analysis also resulted in one inductive sub-category: *Prerequisites for Intervention Interaction* (Table 6).

#### Interactional workability

The intervention was described as a tool for people with dementia and informal caregivers to facilitate daily structure and routines. However, some informal caregivers raised the importance of finding the right balance and not being overly ambitious when seeking to operationalize the intervention in everyday life, to minimize the risk of the person believing they are failing:

*“You must choose a level where you know 100% that the person will succeed, instead of stepping up too much”* (Informal caregiver 19)

Similarly, one person with dementia emphasized that maintaining discipline to engage with the intervention may be challenging, particularly amid the demands and responsibilities in everyday life with dementia. They reflected on the importance of perceiving the intervention as a help and sense of security, rather than an additional burden:

*“Discipline. That’s probably the hard part right now. Not letting it* [the intervention]* … How should I put it, just fade into the grey everyday life, and there’s already enough misery to deal with, and now I’m supposed to handle this* [the intervention] *too? But it should always feel like it’s a help, and I’m sure it will once you get into it. It’s… help and security, I guess that’s how you could summarize it”* (Person with dementia 7)

#### Relational integration

Informal caregivers and people with dementia articulated confidence that some professionals, e.g., dementia/specialist nurses, were suitable intervention guides due to their dementia competence and knowledge, which would facilitate continued intervention engagement. However, other workforces, e.g., general practitioners, assistant nurses, and volunteers, were considered unsuitable:

*“I believe more in nurses, because they have more contact with patients. General practitioners are maybe a little too focused on FASS* [information service about pharmaceuticals]*”* (Informal caregiver 10)

#### Skill-set workability

A need for intervention guides to have appropriate healthcare education was raised as important. However, professional experience of working with dementia was perceived as most important:

*“Not so much education as experience”* (Informal caregiver 15)

Lived experience of being an informal caregiver was also considered a potential facilitator. One informal caregiver highlighted the importance of intervention training for intervention guides providing guidance to the dyad. Another informal caregiver voiced a need for dementia education for informal caregivers before starting to support the person with dementia to use the intervention:

*“Informal caregivers probably need* [education] *in the beginning… To get an understanding of dementia, without this intervention. Start by getting education about the different dementia subtypes that exist, how to respond, what can you expect”* (Informal caregiver 19)

#### Contextual integration

Some people with dementia and informal caregivers mentioned the need for additional resources in terms of *“time”* (Person with dementia 1), funding, and staffing for successful implementation. In particular, lack of time among informal caregivers was highlighted as a barrier to using the intervention:

*“You must have plenty of time”* (Informal caregiver 6)

#### Prerequisites for intervention interaction

Common dementia symptoms were described as potential barriers to intervention engagement. Informal caregivers perceived it important to consider day-to-day fluctuations in cognitive abilities and mood due to dementia. For example, some days may be more suitable for working with the intervention than others, making the timing and flexibility of intervention delivery important:

*“It has to do with the daily form, so you have to be very ’here and now’. It might not work one day, then you simply have to do it the next day”* (Informal caregiver 3)

Being motivated, disciplined, having good within-dyad communication, and the person with dementia being ready to make a change were considered facilitators for intervention engagement:

*“I also think that you have to be ready and have... a will within yourself”* (Person with dementia 8)

## Discussion

Using NPT [27], we gained insights into the: (1) perceptions of people with dementia and informal caregivers regarding the intervention compared to existing practices (*Coherence*), (2) relational work required to foster individual and collective adoption and maintenance of the intervention in routine practice (*Cognitive Participation*), and (3) work that must be undertaken to integrate the intervention into routine practice (*Collective Action*).

Findings indicated strong *Coherence* among people with dementia and informal caregivers, where they clearly differentiated this intervention compared to current practice. A psychological treatment gap was identified, alongside a need for interventions to reduce this treatment gap (*Differentiation*). This contrasts with findings from a similar study involving people with dementia, informal caregivers, and healthcare professionals, where some participants struggled to understand how a new model of primary care-based dementia care differed from existing practices [38]. However, this challenge does not appear unique to this population. For example, one study reported similar difficulties among people with type 2 diabetes in making sense of current treatment [39]. A potential explanation for our different findings may relate to the involvement of people with dementia and informal caregivers early in the intervention development phase, rather than feasibility phase or post-implementation. Involvement in this phase required active engagement with and critical assessment of the intervention and its materials. Potentially, this process may have enhanced their ability to differentiate the intervention from existing practices. However, it is important to highlight that the perceived treatment gap was based on the perspectives of people with dementia and informal caregivers in the present study, however, given psychological support provision varies between regions in Sweden [40], findings may not be transferable to other regions. People with dementia and informal caregivers had a shared understanding of the intervention's purpose (*Specification*) and recognized its potential value, benefits, and importance (*Internalization*). However, an important barrier to *Internalization* that was not identified by professionals [19] was the need to find the right balance for intervention engagement, e.g., that being overly ambitious might potentially lead people with dementia to feel they ‘failed’ the intervention. This suggests a need for intervention guides to explain to the dyad that the intervention uses a structured and graded approach to increase activity [14], starting with engaging in activities considered the least difficult to achieve, with intervention guides helping to break down activities further if found to be unmanageable [11].

Findings suggest strong *Cognitive Participation* among people with dementia and informal caregivers. Generally, both groups accepted and expressed willingness to adopt and use the intervention (*Enrolment*). However, they emphasized the importance of receiving face-to-face guidance from the intervention guide. This is consistent with findings from previous research involving informal caregivers of people with dementia, where caregivers similarly express readiness to adopt an intervention aimed at improving outcomes for the person they care for [41]. This high level of engagement may be explained by the emotional and relational motivations that often underpin informal caregiving. A previous review found that caregivers of people with dementia are driven by various interconnected motives, including love, duty, and reciprocity [42], which may translate into willingness to support interventions. However, concerns that the intervention material would overwhelm the dyad, together with cognitive difficulties, were also communicated as barriers to *Enrolment*. To increase implementation potential and reduce treatment burden, minimizing redundant text in the intervention material will be important [18].

Unlike professionals [19], people with dementia and informal caregivers did not express a need for key interest-holders (e.g., managers) to drive the intervention forward (*Initiation*). This suggests professionals may focus on barriers and facilitators related to macro level (i.e., factors related to policymakers/governments) and meso level (i.e., factors related to healthcare services), whereas people with dementia and informal caregivers focus on micro level (i.e., factors related to patients/caregivers), further stressing the importance of involving all members of the healthcare triad (professional-patient-caregiver) during intervention development. While people with dementia and informal caregivers generally acknowledged intervention relevance, some articulated reservations about its suitability, e.g., for people with severe dementia (*Legitimation*). People with dementia and informal caregivers also stressed the importance of establishing and maintaining a trusting relationship with the intervention guide (*Relational Interaction*). This finding is in line with previous research, where establishing a trusting and consistent care relationship is essential for continuous care and may overcome barriers to accessing and using formal care [43].

Further relating to *Cognitive Participation*, viewing dementia acceptance as a prerequisite for recognizing a need for the intervention (*Definition and Evaluation of the Situation*) was a finding similar to professionals [19], and dementia acceptance has been reported in previous research as a common barrier for accessing and using care [43]. Lack of dementia acceptance (i.e., denial) is especially common in the early stages [44]. Given that the intervention is developed for people with mild-to-moderate dementia [14], denial may, therefore, present a challenge for intervention engagement. Those endorsing and guiding the intervention will require training in how to support these people with dementia who are experiencing difficulties accepting their diagnosis. This finding is not unique to dementia. Similar challenges have been observed in other conditions, for example, people with type 2 diabetes also describe diagnosis acceptance as essential for engaging with treatment [39]. Similarly, in bipolar disorder, acceptance of the diagnosis has been shown to support the transition into treatment [45]. This suggests that across conditions, psychological readiness to engage may depend on diagnosis acceptance. Lack of incentives for informal caregivers due to the progressive nature of dementia was also identified as a barrier. This finding suggests a need for people with dementia and informal caregivers to receive information that the intervention aims to increase activity, and therefore may decrease depressive symptoms and increase overall health and quality of life [46]. The intervention may also improve caregiver well-being [47] via improved functioning in the person with dementia [6]. However, if the informal caregiver experiences high levels of burden and poor psychological well-being, involving a different informal caregiver to support the person with dementia may be necessary [14]. Using implementation strategies such as developing and distributing educational materials for informal caregivers to learn about the intervention and potential benefits will be important to overcome these barriers [48].

*Collective Action* was demonstrated in several ways, including seeing the intervention as a tool to facilitate the establishment of routines in everyday life (*Interactional Workability*) and having confidence that dementia/specialist nurses have the competence to provide guidance (*Relational Integration*). The importance of intervention guides having knowledge of and experience of working with dementia was articulated (*Skill-Set Workability*). Adopting implementation strategies related to educating and training intervention guides to facilitate the development of appropriate dementia knowledge and competencies when implementing the intervention will be important, e.g., education, ongoing training and supervision, and train-the-trainer strategies [48]. The need for additional implementation resources was mentioned (*Contextual Integration*), although professionals emphasized this aspect more [19]. This finding also aligns with wider literature, where professionals consistently report a need for additional resources, including time, funding, and staff, to support the successful implementation of interventions [39, 49]. Furthermore, informal caregivers stressed how day-to-day fluctuations resulting from dementia could significantly impact intervention engagement (*Prerequisites for Intervention Interaction*). Previous research has reported dementia symptoms as common barriers to activity engagement [50], and lack of motivation as a barrier to intervention use [51]. Strategies to increase motivation in the person with dementia may include: (1) carefully selecting times of the day when experiencing increased energy/motivation, (2) working with the intervention little at a time and often, and (3) providing encouragement to increase positive reinforcement [14].

### Limitations

Our study has several limitations. First, the transferability of findings may be limited given that: (1) people with dementia and informal caregivers were predominantly residing in Central Sweden, (2) no ethnic minority groups were recruited, and (3) people with dementia were not required to meet diagnosis for depressive disorder. Second, interviewers had limited prior experience conducting interviews with people with dementia, which may have affected the depth of the data collected from this group. Additional training on communication strategies and interview techniques specific for this group may have improved the richness of the data [52]. Related to this, interview guides were similar to those used with professionals. Given that NPT was developed based on studies in healthcare settings [27], language may have been difficult for non-professionals to understand. Whilst NPT recognizes patients as active agents as opposed to passive recipients, it has been criticized for lacking consideration of the patient perspective [53], and traditionally, patients are not as centrally involved in implementation as professionals [53]. Improved adaptation of interview guides via involving the Public Advisory Group may have improved understanding and potentially generated richer data. Third, as reported in previous research, coding was not always straightforward due to conceptual overlaps between constructs [29]. Similarly, some data relevant to implementation potential were not captured by NPT, and difficulties in coding data falling outside NPT are reported elsewhere [54]. To overcome this limitation, we included inductive sub-categories.

## Conclusions

Together with people with dementia and informal caregivers, we successfully identified barriers and facilitators for future implementation of a guided low-intensity BA intervention for people with dementia and depression within the Swedish context. Additional implementation barriers and facilitators were identified to those recognized by professionals [19], especially at the micro level. This emphasizes the importance of involving the patient-caregiver dyad in developing and implementing complex interventions, especially in the context of self-management, where involving patients and families is critical for successful implementation [21]. Identified barriers and facilitators will inform intervention adaptations and the adoption of evidence-based implementation strategies [55]. A subsequent pragmatic feasibility study will be conducted to further explore the acceptability and feasibility of the intervention and study procedures, alongside implementation potential.

## Supplementary Information

Additional file 1. Standards for reporting qualitative research checklist.

Additional file 2. Interview guides informed by NPT.

## Data Availability

The datasets generated and/or analyzed during the current study are not publicly available due to privacy or ethical restrictions but are available from the corresponding author on reasonable request.

## Abbreviations

BA: Behavioral Activation
NPT: Normalization Process Theory
