# Supplementary material for: People with dementia and informal caregivers’ perceptions of barriers and facilitators to implementing a behavioral activation intervention: a qualitative study using Normalization Process Theory
Source: BMC Geriatr. 2025 Jun 11;25:424. doi: 10.1186/s12877-025-06087-1 (PMC12160347; doi:10.1186/s12877-025-06087-1)
Supplement: Supplementary file 2 — Additional file 2. Interview guides informed by NPT. [file 12877_2025_6087_MOESM2_ESM.docx]

**Interview guide informed by Normalization Process Theory**

**(Informal caregivers)**

1. What are your first impressions of the intervention?
2. How would you describe the purpose of the intervention?
3. How does the support provided in the intervention differ from the support people with memory difficulties are currently receiving?
    a. How does the support provided in the intervention differ from the support you are currently receiving?
    b. What support would you like for your own well-being?
4. What impact do you think the intervention can have on people with memory difficulties?
5. What impact do you think the intervention can have on informal caregivers?
6. What type of support do you think people with memory difficulties need to understand what the intervention is and how it should be used?
    a. What difficulties might people with memory difficulties experience when using the intervention?
7. What type of guidance do you think informal caregivers need to understand what the intervention is and how it should be used?
    a. What difficulties might informal caregivers experience when using the intervention?
8. There are currently no trained healthcare group who can provide intervention guidance to informal caregivers and people with memory difficulties in Sweden. Who or which group do you think is best at providing the guidance?
9. There are different ways of providing guidance. For example, via telephone, e-mail, face-to-face, or group settings. Which way do you think is the best for providing guidance?
   1. Which way of receiving guidance would you prefer?
10. If the guidance is provided face-to-face, where do you think the guidance should take place?
11. How often do you think informal caregivers would need guidance in using the workbook?
    1. How often would you need guidance in using the workbook?
12. How would the intervention impact the support informal caregivers currently provide to people with memory difficulties?
    1. How would the intervention impact the support you currently provide to a person with memory difficulties?

**Interview guide informed by Normalization Process Theory**

**(People with dementia)**

1. What are your first impressions of the intervention?
2. How would you describe the purpose of the intervention?
3. How does the support provided in the intervention differ from the support people with memory difficulties are currently receiving?
    a. How does the support provided in the intervention differ from the support you are currently receiving?
4. What impact do you think the intervention can have on people with memory difficulties?
5. What impact do you think the intervention can have on informal caregivers?
6. What type of support do you think people with memory difficulties need to understand what the intervention is and how it should be used?
    a. What difficulties might people with memory difficulties experience when using the intervention?
7. What type of guidance do you think informal caregivers need to understand what the intervention is and how it should be used?
    a. What difficulties might informal caregivers experience when using the intervention?
8. There are currently no trained healthcare group who can provide intervention guidance to informal caregivers and people with memory difficulties in Sweden. Who or which group do you think is best at providing the guidance?
9. There are different ways of providing guidance. For example, via telephone, e-mail, face-to-face, or group settings. Which way do you think is the best for providing guidance?
   1. Which way of receiving guidance would you prefer?
10. If the guidance is provided face-to-face, where do you think the guidance should take place?
11. How often do you think people with memory difficulties would need support in using the workbook?
    1. How often would you need support in using the workbook?
12. How would the intervention impact the support people with memory difficulties currently receive from informal caregivers?
    1. How would the intervention impact the support you currently receive from an informal caregiver?
